# Effect of Thermal Processing on Surface Roughness of Injection-Molded Denture Base Polymers

**DOI:** 10.3390/polym18091010

**Published:** 2026-04-22

**Authors:** Bozhana Chuchulska, Mariya Dimitrova, Boyan Dochev, Kliment Georgiev

**Affiliations:** 1 Department of Prosthetic Dentistry, Faculty of Dental Medicine, Medical University of Plovdiv, 4000 Plovdiv, Bulgaria; bozhana.chuchulska@mu-plovdiv.bg; 2 Department of Mechanics, Faculty of Mechanical Engineering, Technical University of Sofia, Branch Plovdiv, 4000 Plovdiv, Bulgaria; 3 Center of Competence “Smart Mechatronic, Eco-and Energy-Saving Systems and Technologies”, 4000 Plovdiv, Bulgaria; 4 Department of Mechanical and Instrument Engineering, Faculty of Mechanical Engineering, Technical University of Sofia, Branch Plovdiv, 4000 Plovdiv, Bulgaria; k.georgiev@tu-plovdiv.bg

**Keywords:** removable dentures, surface features, dental materials, polyamides, testing, prosthodontics

## Abstract

Surface roughness and mechanical performance are critical determinants of the clinical behavior, hygiene, and longevity of denture base materials. This study investigated the influence of two extrusion temperatures—280 °C and 300 °C—on both the surface roughness and compressive strength of ThermoSens thermoplastic polymer specimens over a 7-day immersion period. Surface roughness was evaluated at baseline, 24 h, and 7 days using a contact profilometer, while compressive strength was measured after 7 days following ISO 604 guidelines. Samples processed at 300 °C exhibited a significantly greater reduction in surface roughness over time (28.3%) compared with those processed at 280 °C (18.3%). However, although specimens processed at 300 °C showed a greater percentage reduction, their absolute roughness values remained higher than those processed at 280 °C. Compression testing demonstrated higher strength and modulus values in the 300 °C group (91.6 ± 1.8 MPa; 1887.9 ± 42.3 MPa) compared to the 280 °C group (82.3 ± 2.1 MPa; 1755.4 ± 38.7 MPa). These findings indicate a trade-off between improved mechanical performance at higher processing temperatures and lower surface roughness at lower temperatures, highlighting the need for the careful optimization of processing conditions.

## 1. Introduction

The surface properties of denture base materials, particularly surface roughness, are critical determinants of their clinical performance, hygiene, and long-term durability [1]. Increased surface roughness facilitates microbial adhesion and biofilm formation, significantly elevating the risk of denture-related stomatitis and other inflammatory conditions of the oral mucosa [2,3]. Microorganisms such as Candida albicans and various bacterial species readily colonize irregular denture surfaces, forming structured biofilms that exhibit increased resistance to mechanical and chemical cleaning methods [4]. The persistence of such biofilms represents a major clinical challenge, particularly in elderly and immunocompromised patients [4,5,6].

From a physicochemical perspective, surface irregularities provide protected niches that favor microbial retention and proliferation while limiting the effectiveness of routine hygiene procedures [7,8]. In addition to these biological implications, surface roughness also influences the mechanical interaction between the denture base and oral tissues. Rougher surfaces can increase friction, contribute to mucosal irritation, and negatively affect patient comfort, retention, and prosthesis stability [9,10]. Over time, these effects may compromise both patient compliance and the functional lifespan of the prosthetic appliance.

Beyond biological considerations, surface quality is closely linked to the internal structure and processing history of the material. In thermoplastic denture base polymers, surface roughness is not only a result of finishing procedures but is also significantly influenced by manufacturing parameters [11,12]. Factors such as processing temperature, injection pressure, cooling rate, and mold characteristics affect polymer chain mobility, degree of fusion, and the formation of internal defects, including voids and gas inclusions [13,14]. These structural features, in turn, determine both surface morphology and mechanical behavior.

Processing temperature is one of the most influential parameters in the fabrication of thermoplastic polymers. It governs melt viscosity, flow behavior, and the ability of the material to fully adapt to the mold geometry [15]. At lower temperatures, insufficient polymer flow and incomplete chain mobility may result in poor mold filling and structural heterogeneity. Conversely, higher processing temperatures can enhance polymer chain diffusion and fusion, improving mechanical properties, but may also promote gas entrapment and the formation of microvoids, potentially affecting surface characteristics [16,17]. Therefore, identifying an optimal thermal processing window is essential for balancing surface quality and mechanical performance.

Thermoplastic polyamides, such as ThermoSens, are increasingly used as monomer-free denture base materials due to their favorable properties, including flexibility, impact resistance, and reduced risk of allergic reactions compared to conventional polymethyl methacrylate (PMMA) [18]. However, these materials typically exhibit lower stiffness and are more sensitive to processing conditions, making the optimization of fabrication parameters particularly important for ensuring adequate mechanical strength and long-term clinical reliability [19,20].

Although previous studies have investigated surface modification techniques, polishing protocols, and microbial adhesion in denture base materials, there remains a relative lack of data regarding the direct influence of thermal processing conditions on both surface roughness evolution and mechanical properties in thermoplastic systems [21]. Moreover, most studies evaluate these properties independently, whereas in clinical practice they are closely interrelated and jointly determine the overall performance of the prosthesis [22,23].

The present study addresses this gap by evaluating the effect of two processing temperatures―280 °C and 300 °C―on the surface roughness and compressive strength of ThermoSens thermoplastic polymer specimens. These temperatures were selected as controlled deviations around the manufacturer-recommended value to assess the influence of thermal processing on material behavior.

However, limited data exist on how thermal processing parameters influence both surface roughness evolution and mechanical performance of monomer-free thermoplastic denture materials. This study aims to provide a comprehensive evaluation of how processing temperature influences both surface characteristics and mechanical properties, thereby contributing to the optimization of fabrication protocols for thermoplastic denture base materials.

## 2. Materials and Methods

### 2.1. Experimental Sample Preparation

Thermoplastic polymer specimens (ThermoSens, Vertex-Dental B.V., Soesterberg, The Netherlands) were fabricated using a standardized injection molding protocol at two processing temperatures: 280 °C and 300 °C. These temperatures were selected as controlled deviations below and above the manufacturer-recommended processing temperature of 290 °C. This approach enables evaluation of the effect of reduced versus increased thermal energy on polymer flow behavior, chain mobility, and potential gas entrapment during processing.

All specimens were produced using the same injection molding equipment under identical processing conditions, including injection pressure, cooling rate, and material batch, in order to minimize variability. Following fabrication, specimens were allowed to cool under controlled ambient conditions to prevent thermal distortion.

Post-processing included removal of excess material and verification of specimen geometry in accordance with the requirements for profilometric and mechanical testing. All samples were visually inspected for defects such as voids, incomplete filling, or warping, and any defective specimens were excluded from further analysis.

For each temperature condition, three specimens were produced per batch, and the experiment was repeated three times, resulting in a total of nine specimens per group (*n* = 9). All specimens were randomly coded to ensure blinding during subsequent measurements.

For aging procedures, samples were immersed in distilled water at 37 °C in sealed containers to simulate intraoral conditions. The immersion medium was refreshed every 24 h. Surface roughness measurements were performed at baseline (prior to immersion), after 24 h, and after 7 days.

### 2.2. Surface Roughness Testing

Surface roughness was evaluated using a contact profilometer (INSIZE ISR-C002, INSIZE Co., Ltd., Suzhou, China), equipped with a diamond stylus. Prior to measurements, the device was calibrated using a certified reference standard (Ra = 1.2 µm).

Measurement parameters were standardized as follows:

- Measurement range: 0.4–3.2 µm.
- Cut-off length (λc): 0.8 mm.
- Evaluation length: 1.6 mm (2λc).
- Stylus speed: According to manufacturer specifications.

Each specimen was measured at three different, parallel locations to account for surface variability. The mean Ra value and standard deviation were calculated for each specimen at each time point.

All measurements were performed by a single calibrated operator who was blinded to the processing temperature of the samples.

### 2.3. Compression Testing

Compression testing was conducted after 7 days of immersion using a universal testing machine (Instron 3369, Instron Corp., Norwood, MA, USA), in accordance with ISO 604 standards for compressive properties of polymeric materials.

Cylindrical specimens (*n* = 9 per group) were positioned vertically between compression plates and subjected to a uniaxial load at a crosshead speed of 1 mm/min until failure or the occurrence of a clear yield point.

The following parameters were recorded:

- Compressive strength (MPa).
- Compressive modulus (MPa).

Mean values and standard deviations were calculated for each group. Testing was performed by an operator blinded to the processing conditions.

### 2.4. Statistical Analysis

Statistical analysis was performed using repeated-measures one-way analysis of variance (ANOVA) to evaluate changes in surface roughness over time within each temperature group. When significant differences were identified, Bonferroni post hoc tests were applied for pairwise comparisons.

Differences in compressive strength and modulus between the two temperature groups were analyzed using independent-samples *t*-tests. A significance level of *p* < 0.05 was adopted for all statistical analyses.

## 3. Results

### 3.1. Surface Roughness Testing Results

The surface roughness (Ra) of ThermoSens polymer specimens was evaluated at three time points―baseline, 24 h, and 7 days―for both processing temperatures (Table 1).

For specimens processed at 300 °C, the initial surface roughness was 2.750 ± 0.030 µm. A slight decrease was observed after 24 h (2.697 ± 0.021 µm), followed by a more pronounced reduction after 7 days (1.972 ± 0.014 µm), corresponding to an overall decrease of 28.3%. The detailed values are presented in Table 1.

In contrast, specimens fabricated at 280 °C exhibited lower initial surface roughness values (1.593 ± 0.035 µm) (Table 2). After 24 h, roughness decreased to 1.475 ± 0.028 µm and further to 1.301 ± 0.022 µm after 7 days, representing an overall reduction of 18.3%. These results are summarized in Table 2.

Although specimens processed at 300 °C demonstrated a greater percentage reduction in surface roughness over time, their absolute roughness values remained higher than those of specimens processed at 280 °C at all evaluated time points.

**Figure 1:**
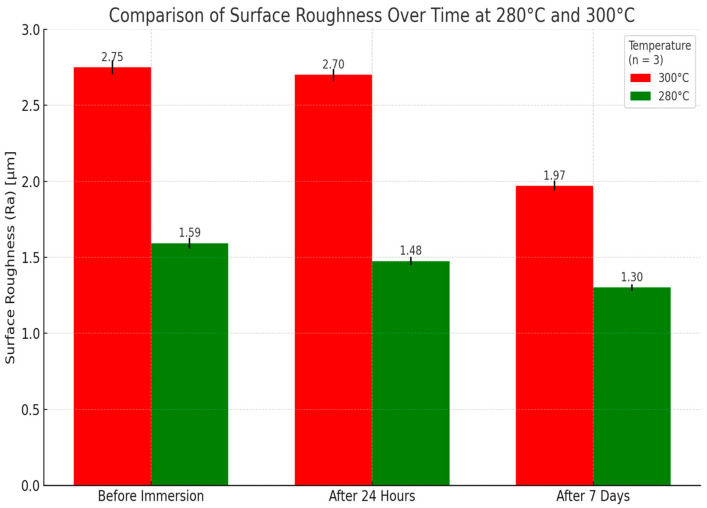
Surface roughness (Ra) changes over time for samples processed at 280 °C and 300 °C.

The progressive decrease in surface roughness for both temperature groups over time is shown in Figure 1.

Figure 1 illustrates the progression of surface roughness (Ra) in ThermoSens polymer samples processed at 280 °C and 300 °C over the three evaluation intervals. As shown, both temperature groups exhibited a consistent reduction in Ra values from baseline through 24 h and after 7 days of immersion. Although specimens processed at 300 °C exhibited a greater relative reduction in surface roughness over time, their absolute roughness values remained consistently higher than those of the 280 °C group at all evaluated time points. Statistical analysis using repeated-measures ANOVA revealed that the reductions in surface roughness were significant within each group (*p* < 0.05). Post hoc Bonferroni comparisons confirmed significant differences between baseline and 24 h, as well as between 24 h and 7 days, for both temperature conditions.

**Figure 2:**
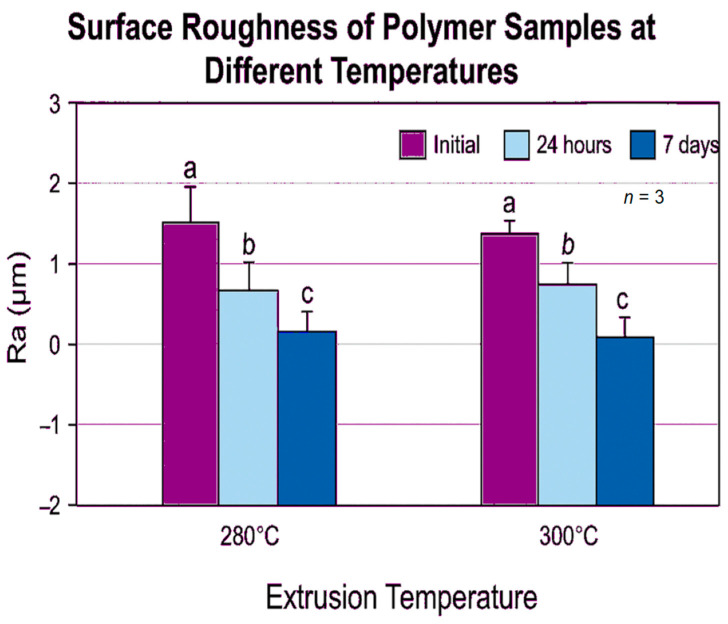
Surface Roughness of ThermoSens Samples at 280 °C and 300 °C Across Time Points: (a) Initial phase (baseline), (b) after 24 hours, (c) after 7 days.

The comparative surface roughness values and statistical differences between groups are illustrated in Figure 2.

These results indicate that both processing temperatures lead to a reduction in surface roughness over time; however, higher temperature processing results in a greater relative decrease, while lower temperature processing maintains lower absolute roughness values.

### 3.2. Compression Test Results

Compression testing was performed after 7 days of immersion to evaluate the effect of processing temperature on mechanical performance.

Specimens processed at 300 °C demonstrated higher compressive strength and modulus values compared to those processed at 280 °C (Table 3). The mean compressive strength was 91.6 ± 1.8 MPa for the 300 °C group and 82.3 ± 2.1 MPa for the 280 °C group. Similarly, the compressive modulus was 1887.9 ± 42.3 MPa at 300 °C and 1755.4 ± 38.7 MPa at 280 °C. The detailed results are presented in Table 3.

Statistical analysis using independent t-tests confirmed that the differences in compressive strength and modulus between the two groups were significant (*p* < 0.05).

These findings indicate that increasing the processing temperature enhances the mechanical performance of the thermoplastic polymer, resulting in increased resistance to compressive loading.

## 4. Discussion

The present study investigated the influence of processing temperature on the surface roughness and mechanical performance of ThermoSens thermoplastic denture base material. The observed differences between the two temperature conditions confirm that even moderate deviations from the manufacturer-recommended processing temperature significantly influence both surface characteristics and mechanical properties.

The results demonstrated that specimens processed at 280 °C exhibited lower absolute surface roughness values at all evaluated time points compared to those processed at 300 °C. This finding suggests that lower processing temperatures may promote more stable surface formation during injection molding, potentially due to reduced thermal degradation and lower levels of gas entrapment [21,22]. In contrast, the higher initial roughness observed at 300 °C may be associated with increased melt flow and enhanced interaction with the mold surface, as well as the possible formation of microstructural irregularities during cooling [23,24].

Despite these differences in absolute values, specimens processed at 300 °C exhibited a greater relative reduction in surface roughness over time. After 7 days of immersion, the reduction in roughness reached 28.3% at 300 °C compared to 18.3% at 280 °C. This behavior may be associated with increased interaction between the material and the immersion medium, potentially facilitated by subsurface structural features. However, since porosity and internal defects were not directly measured in the present study, such explanations remain hypothetical and should be interpreted with caution [25,26,27]. The reduction in surface roughness observed in both groups after immersion is consistent with previous studies reporting fluid sorption effects in thermoplastic dental polymers. Water uptake can lead to partial filling of surface irregularities and microvoids, resulting in a smoother surface profile over time [28,29]. Similar trends have been described in polyamide-based materials, where the most significant changes occur within the first week of immersion [30].

From a mechanical perspective, specimens processed at 300 °C demonstrated significantly higher compressive strength and modulus compared to those processed at 280 °C. This improvement is likely related to enhanced polymer chain mobility and more complete fusion during injection molding at elevated temperatures. Increased thermal energy facilitates chain diffusion and intermolecular bonding, leading to improved structural integrity and resistance to deformation under load [31,32]. These findings are in agreement with previous studies demonstrating that higher processing temperatures can improve the mechanical performance of thermoplastic polymers, albeit sometimes at the expense of surface quality [33,34,35].

The present study has several limitations. Firstly, the internal structure of the material, including porosity and potential gas inclusions, was not directly evaluated using techniques such as micro-computed tomography or density analysis. As a result, interpretations regarding the relationship between processing temperature and internal heterogeneity remain speculative. Secondly, although the sample size *(n* = 9 per group) is consistent with similar experimental studies, it limits the statistical power and generalizability of the findings. Future studies incorporating larger sample sizes and direct structural characterization methods are recommended to confirm and extend these observations.

The relationship between processing temperature and material performance highlights a trade-off between surface quality and mechanical properties. Lower temperatures favor smoother initial surfaces, while higher temperatures enhance mechanical strength and promote greater relative surface smoothing over time. These findings emphasize the importance of optimizing processing parameters to achieve a balance between hygiene-related surface characteristics and functional durability [36,37,38].

It is important to note that some of the interpretations regarding internal structure, such as porosity and gas entrapment, are based on indirect observations rather than direct measurements [39,40]. Therefore, these explanations should be considered plausible mechanisms rather than definitive conclusions. Future studies should incorporate direct characterization techniques, such as micro-computed tomography or density analysis, to better elucidate the relationship between processing temperature and internal material structure.

## 5. Conclusions

A higher processing temperature (300 °C) improved the compressive strength and modulus of the material, but resulted in higher absolute surface roughness compared to processing at 280 °C. Despite a greater relative reduction in roughness over time, this indicates a trade-off between mechanical performance and surface quality. Within the limitations of this study, including the absence of direct porosity measurements and the relatively small sample size, these findings should be interpreted with caution. Further research is needed to better understand the relationship between processing conditions, internal structure, and long-term material behavior.

## Figures and Tables

**Table 1:** Surface Roughness (Ra) of Polymer Samples at 300 °C.

Time Point	Mean Ra (µm)	Standard Deviation (±µm)	% Reduction from Initial
Before Immersion	2.750	±0.030	–
After 24 h	2.697	±0.021	1.9%
After 7 Days	1.972	±0.014	28.3%

**Table 2:** Surface Roughness (Ra) of Polymer Samples at 280 °C.

Time Point	Mean Ra (µm)	Standard Deviation (±µm)	% Reduction from Initial
Before Immersion	1.593	±0.035	–
After 24 h	1.475	±0.028	7.4%
After 7 Days	1.301	±0.022	18.3%

**Table 3:** Compressive Strength and Modulus of ThermoSens Specimens After 7 Days of Immersion.

Temperature (°C)	Compressive Strength (MPa)	Standard Deviation (MPa)	Compressive Modulus (MPa)	Standard Deviation (MPa)
280 °C	82.3	±2.1	1755.4	±38.7
300 °C	91.6	±1.8	1887.9	±42.3

## Data Availability

The original contributions presented in this study are included in the article. Further inquiries can be directed to the corresponding authors.
